# Understanding the struggle: Unique challenges of adherence in male diabetic patients in Tshwane

**DOI:** 10.4102/safp.v66i1.5998

**Published:** 2024-09-30

**Authors:** Refilwe S.N. Mokoena, Eugene M. Makhavhu, Livhuwani Tshivhase

**Affiliations:** 1 Department of Nursing Science, School of Health Care Sciences, Sefako Makgatho Health Sciences University, Tshwane, South Africa; 2 Department of Health Studies, School of Social Sciences, College of Human Sciences, University of South Africa, Tshwane, South Africa

**Keywords:** adherence, diabetes treatment, male diabetic patients, type 2 diabetes mellitus, well-being, public health, health issues, healthcare

## Abstract

**Background:**

Type 2 diabetes prevalence is steadily increasing worldwide, and South Africa is one of the countries in Africa with the highest prevalence of this disease, along with other non-communicable diseases. The adherence to treatment in male patients with type 2 diabetes is influenced by their attitudes towards medication and how they perceive their condition. To some extent, these factors impact the treatment outcomes for patients undergoing type 2 diabetes treatment. The purpose of this study was to investigate the perceptions of male patients with type 2 diabetes on their adherence to diabetic therapy. The study was conducted in the clinics of the City of Tshwane Metropolitan municipality in Gauteng.

**Methods:**

This study followed a qualitative, exploratory design. Data were gathered from 15 male patients who were purposefully sampled through in-person, one-on-one interviews with the principal investigator. The eight steps outlined by Tesch were used to analyse the participant data.

**Results:**

Emergent themes indicated that there were barriers to adherence to diabetic treatment and also factors that promoted adherence to diabetic treatment among the participants. Several factors were found to affect treatment uptake among the participants.

**Conclusion:**

Patients demonstrated various reactions to diabetic treatment, highlighting the need for reinforcing education at the time of diagnosis and treatment initiation. Additionally, regular patient follow-up may be essential to improve adherence among patients.

**Contribution:**

The study highlights the importance of health promotion and the need to develop materials for medication-specific counselling for patients receiving diabetic treatment, in order to promote adherence.

## Introduction

Diabetes mellitus is a chronic medical disorder marked by elevated blood glucose or blood sugar levels. Over time, this condition can cause significant harm to various organs, including the heart, blood vessels, kidneys, eyes and nerves.^1^ The highly prevalent type 2 diabetes interrupts the body’s ability to use insulin to control blood sugar levels and alters how the body uses sugars for energy.^1^ The condition is among the non-communicable diseases that contribute to South Africa’s burden of diseases. It is also impacting younger generations and in a 2021 study, there was an estimate of approximately 41 600 new cases of type 2 diabetes among children and adolescents worldwide.^2^

Public health is challenged by the exponential increase in diabetes diagnoses, with a total of 537 million people worldwide living with the disease,^3^ and additional increases in prevalence predicted. From 4.5% in 2010 to 12.7% in 2019, the prevalence of diabetes mellitus has quickly increased in South Africa.^4^ Diabetes is a primary cause of mortality for women and ranks second overall in South Africa’s population’s underlying causes of death.^5^ Although it is a leading cause of death globally and presents serious risks to one’s health and life expectancy, the disease can still be manageable.

Management of diabetes mellitus includes lifestyle modification in the onset of pre-diabetes,^6^ an intermediate stage between normal glycaemia and diabetes. Additional strategies for managing the illness include cutting back on calories, exercising, losing weight and taking hypoglycaemic drugs. Patients with type 2 diabetes are likely to lead normal lives if their condition is well managed.^7^ Effective diabetes care also depends on patient-related aspects, such as self-control, support and treatment adherence, which is when the patient’s behaviour coincides with medical advice. Males suffering from the condition, however, have reportedly been known to worry about changes in their body image, such as weight reduction, which are associated with the condition.^8^ This may frequently hinder treatment adherence. As a result, they stress about being laughed at and not being able to perform daily activities that they previously could, such as sporting activities. This can also lead to unfavourable attitudes about the treatment plan and have an impact on patient’s adherence to treatment.^8^

When patients are adhering to their medications, they should be taking their medication in accordance with medical advice or as they were prescribed. This is measured by the patient’s taking at least 80% of the prescription pills given by their doctor at the same time.^9^ Adherence to diabetes therapy is crucial to achieving treatment effect^10^ and has been linked to improved metabolic control and a higher quality of life. It is a critical factor in preventing complications and improving overall health outcomes. Adherence to treatment and medications may, however, differ from patient to patient and also between males and females. While diabetes affects both men and women, men often face distinct challenges in managing their condition and adhering to prescribed treatment as compared to women.

Men and women view and relate to health differently, particularly in cultural and societal contexts.^11^ Men are generally less likely to seek preventative and curative healthcare services compared to women. They are more likely to be non-users of primary health care. These differences are reflected in the ways that men and women seek health care.^12^ Their health-seeking behaviour may therefore have a direct impact on the management of certain conditions and their treatment outcomes. Cultural, religious, societal and personal factors can also impact a patient’s commitment to maintaining a healthy lifestyle as well as their perceptions, healthy beliefs and attitudes towards treatment strategies for diabetes mellitus.^13^

### Aim

The aim of this study was to explore how male patients with type 2 diabetes perceived their adherence to diabetic therapy in City of Tshwane clinics.

## Research methods and design

This section outlines the methodological approaches employed in this study.

### Study design

This study used a qualitative^14^ research design that was exploratory and descriptive^15^ in nature. The design was chosen for its ability to delve deep into the participants’ perceptions, views and experiences as well as enabling researchers to understand the participants’ viewpoints of the phenomenon under study.^16^ The qualitative design in this study is justified because of the nature of the research problem and the need to understand the depth of the phenomenon from participants who have the experiences. This gives the researcher the ability to capture nuanced and complex experiences and perceptions that influence men’s behaviour regarding diabetes treatment. This approach allowed the researchers to explore the underlying reasons, motivations and barriers that affect treatment adherence in a manner that gives voice to the participants and allow them to express their perspectives in their own words. The qualitative approach granted the researcher a more holistic context around the research question and provided depth and richness of the data needed to understand the unique struggles of male diabetic patients and their adherence to treatment.

### Study setting

The study was conducted in the City of Tshwane Metropolitan municipality clinics in the north of the greater Gauteng province, South Africa. Participants were recruited from three selected clinics with a large number of patients on their roll. The selected clinics are nurse-managed and offer services including curative, preventative and chronic treatment. Part of the services offered by these clinics include the management of diabetic patients, and according to the statistics within the city’s health department, the three clinics had the most patients on diabetic treatment at the time of the study with the highest headcount of diabetes mellitus patients estimated at 1415 monthly between October 2021 and December 2021.

### Study population and sampling

The population in this study comprised male diabetic patients who were taking treatment at the selected clinics in the City of Tshwane Metropolitan municipality. Purposive sampling^15^ was used to sample participants, and this was relevant as the researchers aimed to sample participants who fit the study’s inclusion criteria and would be able to respond to the research question. Participants who were diagnosed with type 2 diabetes mellitus (T2DM) and had been on treatment for a period of a year and above were included in the study, while newly diagnosed participants who had recently started on treatment were excluded from participation. A total of 15 persons participated in the study, and data saturation^14^ was used to establish the appropriate sample size. Individual interviews with 11 male participants produced an abundance of data and no new information was realised at the 11th participant. A further four interviews were conducted to confirm data saturation and ensure that there were no gaps in the data gathered. The study therefore concluded with 15 participants.

### Data collection

Data were collected using face-to-face individual interviews. A self-developed interview guide was used to collect the data from the participants. The interview guide was pre-tested with five participants who met the inclusion criteria for participation in one of the clinics. The data from the pre-test is not reported in this study. The interviews were conducted in a private room provided by the facility managers at the selected clinics. To ensure that all that was said by the participants was captured correctly, an audio recorder was used during the interviews. The participants were informed about the recording and had to voluntarily consent to the interviews being recorded prior to the commencement of the interviews. Data collection took place over a period of 5 weeks from the 01 August 2023 and ended on the 06 September 2023. Data were collected by the primary researcher in both Setswana and English languages. While some participants could communicate well in English, others preferred using the local language, Setswana and the primary researcher was fluent in both languages. As a practitioner in the city, the primary researcher applied bracketing to prevent pre-conceived ideas and their influence during data gathering from interfering with participants’ responses.

### Data analysis

Teschs’ approach to thematic analysis was employed in this study.^17^ Audio recordings were listened to and transcribed verbatim by the primary researcher. The transcriptions were read in order to identify relevant features of the data that could be clustered as codes. The codes were collated into potential themes. A comparison of transcripts was done in order to ascertain the common themes, and a clear definition and names were developed for each theme. Sub-themes were also identified to give structure to the emergent themes. An independent coder who is qualified and an expert in the field of qualitative research and holds a Master of Science in Nursing (Msc: Nursing) and Doctor of Literature and Philosophy (DLitt et Phil: Nursing) degree in Nursing was appointed to independently analyse the data. The emergent themes were confirmed and concluded by a consensual discussion with the primary researcher and an independent coder to verify the themes and conclude the analysis.

### Ethical considerations

The study received ethical clearance from the Sefako Makgatho Health Sciences University (reference no.: SMUREC/H/393/2022:PG). Ethical principles of research were followed. Informed consent was sought from participants, and a copy of the information leaflet was shared with participants once information about the research was disseminated to them. To ensure privacy during the data collection process, interviews were held in a private room made available by the clinics, and participants were ensured of their anonymity in participation. During reporting, participants’ identities were kept private, and pseudonyms were used instead. Recorded interviews were kept private and accessible only to the researchers. Participants were made aware of their right to exit the study at any point and that the interviews were being recorded.

Because of the nature of the study, participants were to divulge their medical information that could potentially evoke some emotional responses from participants. The clinical and participants were made aware of such and that they would be supported through the psychological support that the clinic offers on-site. Participants were ensured that their participation in the study would not affect the quality of health service, they would get at the clinic and that they could be free to participate truthfully without fear of being treated unfairly.

## Results

In this section, the findings of the study are presented.

### Socio-demographic data

The study included 15 participants all of which were residents of the City of Tshwane. Of the 15 participants, four were white South Africans while 11 were of African descent. The youngest participant was 21 years of age while the oldest was 74 years old. Treatment durations for participants ranged from 1 year to 17 years (Table 1).

**TABLE 1:** Socio-demographic characteristics of participants.

Participant number	Pseudo names	Age	Employment status	Number of years on diabetic treatment
1	Thapelo	43	Employed	13
2	Moreri	52	Unemployed	15
3	Malema	30	Unemployed	1 year 4 months
4	Mokale	55	Unemployed	7
5	Malete	59	Employed	1
6	Tommy	21	Student	3
7	Mulder	59	Unemployed	17
8	Monare	53	Employed	1
9	Ray	48	Employed	1
10	Terence	39	Unemployed	6
11	Korkie	60	Pensioner	15
12	Curtis	74	Pensioner	15
13	Koos	54	Unemployed	5
14	Radling	55	Employed	1
15	Moabi	59	Self-employed	11

*Source*: Mokoena RSN. Perceptions of male patients regarding adherence to type 2 diabetic treatment in the City of Tshwane clinics, Gauteng Province, South Africa. Masters dissertation, Sefako Makgatho Health Sciences University; 2023

### Overview of themes

This study found two main themes that were supported by eight sub-themes emerging from the data analysis. The emergent themes included the promotion of treatment adherence and barriers to adherence. Table 2 presents the emergent themes and sub-themes.

**TABLE 2:** Themes and sub-themes.

Themes	Sub-theme
1. Barriers to adherence	False belief of complete recovery Therapy-related factors Reluctance for treatment uptake Socio-economic barriers
2. Promotion of adherence to treatment	Fear of symptoms exacerbation Fear of significant complications Healthcare worker’s interventions Minimal clinic visitation

*Source*: Mokoena RSN. Perceptions of male patients regarding adherence to type 2 diabetic treatment in the City of Tshwane clinics, Gauteng Province, South Africa. Masters dissertation, Sefako Makgatho Health Sciences University; 2023

### Theme 1: Barriers to adherence

Participants in this study indicated barriers that play a significant role in them not adhering to diabetic treatment. It was found that after commencing treatment, some participants experienced remission of symptoms and felt better. That subjective feeling of being well led to some participants deciding to stop treatment with the belief that they had recovered. Below are some of the participants’ extracts:

‘My blood sugar levels were consistently normal shortly after I was diagnosed, so I made the independent decision to stop taking my medication.’ (Malema, 30 years old, 16 months on treatment)

‘My blood glucose level was normal, and I felt fantastic. I stopped taking my medication because I was doing well. After stopping, I realized after some time that I was falling ill. I then had to restart the treatment process from scratch. I learned that I should not cut back on my diabetes medication in any way. It is best to follow the doctor’s and nurses’ orders.’ (Terence, 39 year old, 6 years on treatment)

Participants had varying perceptions of diabetic medication. Some perceived the injection to be the preferred option which they would likely adhere to or improve their adherence while others saw the oral medications as the better form of treatment to take, which could improve their adherence:

‘Injection is the fastest and I believe in it. It works better for me than tablets. Although the tablet works well, I would much rather have insulin, which might bring the blood sugar level back to normal quicker.’ (Curtis, 74 years old, 15 years on treatment)

‘I was scared of the injection because they said I should inject myself. This made me not to inject myself as expected, however there was pain on the injection site.’ (Moreri, 52 years old, 15 years on treatment)

‘It would be better for me if I could get an alternative form of treatment. Like pills instead of the injection. At the school residential place when I walk with gents holding a package, I always have to explain [*what is in the package*]. Secondly, we are being searched on entry for drugs. I have to explain myself that I am a diabetic of which I did not want disclose. Pills are easily hidden than the injection.’ (Tommy, 21 years old, 3 years on treatment)

Upon diagnosis, some participants found it difficult to believe they had the condition and were thus in denial. The denial of diagnosis directly impacted participants’ readiness for treatment uptake; thus they were reluctant to start treatment. The reluctance for treatment uptake was also seen by one participant as a direct effect of men’s poor health-seeking behaviour compared to that of women:

‘I was diagnosed with diabetes in 2007. I was reluctant to take treatment initially but I started taking treatment in 2010. This is because I felt I was still very young to inject myself. I also did not understand as they were talking about the injections.’ (Moreri, 52 years old, 15 years on treatment)

‘I was on medication since my diagnosis, but I did not take the treatment as prescribed. I was in denial because I was not so sure I had diabetes. I only realised that during the second episode that this was serious.’ (Moabi, 59 years old, 11 years on treatment)

‘Men are reluctant to visit healthcare facilities. Some believe clinics are meant for women.’ (Mulder, 59 years old, employed, 1 year on treatment)

‘I never experienced problems with my libido like it is said that diabetic patients experience, so I believed I did not have diabetes because I was fine. Most males have denial of diabetes and laugh and tease about it as it is said it affects one’s libido. Because of that, we hide it from our friends. I made my wife aware of my condition and she accepted it.’ (Terence, 39 years old, 6 years on treatment)

Socio-economic-related factors also emerged as a barrier to participants’ adherence to their diabetic treatment. Some socio-economic factors proved to be a hindrance to participants’ access and adherence to treatment:

‘I have previously defaulted on medication for two to three weeks due to challenges with accessing transport to go to the clinic. I struggled with transport and was also not feeling well.’ (Korkie, 60 years old, 15 years on treatment)

‘At some stage I no longer belonged to a medical aid scheme. My prescription was labelled “chronic” and was valid for six months. After the six months I had to go consult a doctor for renewal of script. I never went to consult as my medical aid was terminated. I ended up without medication for one and a half year.’ (Thapelo, 43 years old, 13 years on treatment)

‘When I moved to Nigeria, I discontinued my diabetic treatment for some time because I did not have an income. I did not have a medical aid anymore. In 2021, I then went to collect medication at a state hospital.’ (Koos, 54 years old, 5 years on treatment)

Participants also cited healthcare system-related factors that had a bearing on poor treatment adherence. The long waiting times and long queues at the healthcare facilities were cited as one such barrier:

‘The system of booking appointments is fine, but it lacks the humane factor in it. At times say I am booked for 11h00 and I arrive at 11h30, I am told my appointment time has passed. I am made to wait for everyone who is booked after me to be seen before I am called. Having to wait the whole day without a meal is not good for my health. The rule should be followed but bear in mind you are dealing with people. I had a temptation of leaving without collecting medication because of the long waiting times.’ (Malete, 59 years old, 1 year on treatment)

‘In some in instances I felt like I should leave and go home without getting my medications. My suggestion would be to reduce the number of patients seen per day to reduce the long queues and waiting times.’ (Monare, 53 years old, 1 year on treatment)

### Theme 2: Promotion of treatment adherence

This study also found reasons that would promote participants’ adherence to their diabetic treatment. Some participants indicated the recurrence of symptoms as one situation that prompted them to adhere to treatment:

‘I was getting some symptoms. My bottom lip went numb, and my vision was blurry. Then I realized I started to get thirsty and kept using the toilet frequently to urinate. I recognized the symptoms and that was when I realized that the diabetes had returned. That which led me to go back to the clinic. Only then was I restarted on treatment.’ (Malema, 30 years old, 16 months on treatment)

‘There was something that happened to me after I stopped my medication. I had a tingling and numb sensation on one of my toes. It was very uncomfortable when putting on shoes. Since I got back on medication, this has stopped. Even the ulcer problem, at times I would take a nap and the pain would go. All these have stopped, I believe my body was pushing me to come for treatment. I now have a positive attitude towards my treatment. I always have stock with me even when travelling for funerals, I always carry medication with me.’ (Thapelo, 43 years old, 13 years on treatment)

Participants also indicated some aspects of their lives that assist them to be adherent to medication. Participants reported that their fear of the complications associated with diabetes mellitus influenced their commitment to treatment adherence. The health education received from the nurses as well was highlighted as a role player in participants’ adherence:

‘I don’t want to stop my treatment because I am scared it might cause more damage to my body. I was once in a diabetic coma. Going to the clinic does help me because what is happening to me could be worse. I take my treatment and come to the clinic for survival so that my sugar level can be controlled.’ (Mokale, 55 years old, 7 years on treatment)

‘I was referred to a dietician and the nurses gave me advice. I was told if I don’t take my medication as prescribed, I will have complications like painful feet and blindness. I used to forget. By that time, I was taking it daily. At times I took it at the wrong time as I forgot. But now I am used to my routine of taking medication. I take it as prescribed. I saw the necessity of maintaining a healthy lifestyle like to reduce smoking and reduce alcohol consumption. I have reduced both. Now I have abstained from alcohol for three months.’ (Ray, 48 years old, 1 year on treatment)

Other participants indicated that the less time they are required to spend at the facility, the more adherent they are to treatment. The public-private partnership between government health facilities and private pharmacies has also made it easier for patients to access medications, thereby improving adherence:

‘I come to the clinic every six months to collect my treatment and only come once to see the doctor and have bloods taken. If my glucose level is fine and everything is good, I then get the medicine for the next four months at the Clicks pharmacy closest to me. At the clinic you get the two months follow up and at Clicks you get four months.’ (Korkie, 60 years old, 15 years on treatment)

‘I just to come here and let them see that my glucose level is fine otherwise I cannot come here monthly. The way I got my medication is I come here to get my six months medication. After six months I come for blood collection. The system is good and convenient for me.’ (Radling, 55 years old, 1 year on treatment)

## Discussion

The study findings revealed two main themes that included barriers to adherence and promotion of treatment adherence. This study found that some patients preferred injectable insulin and perceived it as acting faster, while others indicated that they would have preferred oral hypoglycaemic. This had a direct effect on participants’ adherence to treatment. Research from additional studies indicates that patients favoured oral medications over injections.^18^ Research has also revealed that, in order to effectively control diabetes and improve optimal treatment adherence, it is imperative to take the patient’s preferences into account.^19^ Apart from personal preferences, the primary attribute that patients and doctors value most about anti-diabetic medications is their ability to effectively lower blood glucose levels.^20^ Given that there is a potential conflict between patient preferences and treatment efficacy, frank conversations may be required with patients in order to proceed with the most appropriate management.

This study found that participants thought it would be difficult to adhere to insulin therapy because of side effects like injection pain. As a result, they thought taking pills would be a better option. This finding is consistent with another study that found that patients were reluctant to use injectable insulin because of the fear of pain and the unwillingness to inject themselves.^21^ This finding indicated a negative attitude towards injectable insulin therapy because of the immediate effect of pain associated with the route of administration. These results are in line with those of a study conducted in Ethiopia,^22^ which discovered that participants’ adherence to insulin therapy only improved when they displayed positive attitudes towards the treatment. These findings highlight the importance of personalised treatment plans that consider patients’ lifestyle and preference factors that would potentially promote adherence to diabetes treatment. Positive attitudes are associated with higher levels of treatment adherence compared to negative attitudes, according to a different but related study.^23^ The authors go on to argue that attitudes play a role in diabetic patients’ ability to adhere to their diabetic treatment plan and follow doctor’s instructions.^23^

Research has also shown that following a diabetic treatment plan was linked to fewer adverse effects.^24^ Adherence to medications appears to be influenced by side effects as well.^25^ Another study found that T2DM patients are primarily worried about the possibility of side effects from their prescribed anti-hyperglycaemic medications, such as weight loss and gastrointestinal adverse events.^26^ This suggests that patients prioritise the tolerability, ease of use and side-effect profile of oral anti-hyperglycaemic medications. A person-centred, differentiated approach should be used to provide medication adherence education and counselling to patients with diabetes and other chronic conditions, taking into account their unique and varied needs.^25,27^ In this situation, healthcare workers’ communication needs to be enhanced through practising active listening, strengthening patient–provider relationships and encouraging patients to express themselves.^25^

This study found that participants discontinued treatment because of a perceived improvement in their condition. The effectiveness of treatment after some time led to the participants’ assuming that they were in remission, hence the discontinuation of treatment. Misconceptions and a lack of knowledge about the effects of diabetes treatment may be a factor for this. Similar results were seen in other studies where patients with diabetes stopped taking their medication after thinking they had recovered.^28,29^ This misunderstanding of diabetes mellitus as a chronic condition among participants shows the need for improved health education and communication between healthcare providers and patients about the long-term management and effects of mismanagement of diabetes mellitus. In order to overcome the obstacles, nurses and other medical professionals must focus especially on clearing up these misconceptions about diabetes treatment and utilising therapeutic communication to improve patients’ adherence to treatment.^30^

This study found that participants faced socio-economic obstacles to following treatment plans, such as difficulties paying for transportation to the medical facility. Travelling and being overly busy were found to be factors that adversely affected the adherence to taking diabetes medications in a different study.^31^ In their study, Elsous and colleagues discovered a correlation between high levels of adherence and more favourable economic circumstances.^32^ The same study also identified gaps, especially in terms of healthcare resources and disparities in access to care. The results of this study demonstrated that some participants stopped receiving diabetic treatment because they were no longer covered by a medical assistance programme. Similarly, those were the results of a study^33^ that showed low-income and uninsured patients frequently did not follow their prescribed course of care for chronic conditions. Furthermore, another study^34^ revealed that the main reason for non-adherence to diabetic treatment was financial difficulties. In keeping with this, a different study found a positive correlation between medication adherence and health insurance coverage.^35^ Additionally, Dehdari and Dehdari discovered that the cost of insulin, consultation fees with doctors, blood glucose test strips and the absence of health insurance were all significant factors influencing the patient’s adherence to their treatment plans.^36^ This finding underscores the need to consider patients’ socio-economic status and social determinants of health in diabetes management. Furthermore, health system management should explore ways to ensure inclusivity in healthcare and improved access to diabetic care for patients facing economic difficulties to start and retain their treatments.

Some participants newly diagnosed with T2DM felt that they were not ready to start diabetic treatment. However, the cornerstone of successful treatment outcomes is client readiness. Newly diagnosed diabetic patients must take part in an extensive diabetes self-management education programme that includes individualised instruction on diet, exercise, treatment adherence and preventing complications.^37^ This will empower them and help them be more treatment ready. The use of telehealth strategies for educational reinforcement improves patient readiness and facilitates diabetes self-management.^38^

Additionally, the significance of educating, counselling and determining a patient’s readiness for treatment was emphasised. The author posits that it is imperative that newly diagnosed patients with type 2 diabetes receive information that emphasises the condition’s chronic nature, treatment options, the importance of a healthy diet and physical activity regimen, blood glucose monitoring and adherence to diabetic medication. Building patient readiness to start and continue treatment is based on this.^39^

The study participants experienced social stigma as a result of having a chronic illness such as diabetes mellitus. From the viewpoints of the participants, it was also assumed that having diabetes meant that a person was not taking care of themselves. A lot of presumptions also existed regarding loss of libido. It was challenging for participants to tell their friends and family that they have diabetes because of all these presumptions. Treatment adherence might have suffered as a result. Patients with diabetes may feel or see different levels of psychological distress and stigma. Numerous studies have looked into the strong correlation that one study found between psychological distress and the stigma associated with diabetes.^40^ In their study, Browne and colleagues demonstrated how stereotyping – such as obesity, poor eating habits and sedentary lifestyles may have a negative impact on diabetic patients, leading to discrimination from both society and oneself.^41^ Further research on coping mechanisms against stigmatisation is needed, according to another study, as it could advance public education and awareness efforts as well as our comprehension of the stigma associated with diabetes.^42^

Another study posits that people with diabetes who self-stigmatise and do not tell their significant others that they have the disease have lower levels of social support, which has detrimental effect on treatment adherence.^43^ Diabetic individuals frequently worry about how society will view them and whether their lifestyle will be criticised.^41^ It is these assumptions and perceptions that may affect fidelity to diabetic treatment protocols. Studies^44,45^ indicated that stigmatisation and discrimination have a detrimental effect on treatment adherence with other chronic conditions, such as human immunodeficiency virus (HIV). Based on our findings, it would appear that this applies similarly to some patients with T2DM.

The participants in this study believed that a lengthy wait would make it more likely that they would forego diabetic treatment. Toga-Sato states that a key tactic in lowering the rate of diabetes treatment discontinuation is raising patient treatment satisfaction.^46^ Furthermore, as the authors point out, decreasing waiting times is essential to raising patient satisfaction with treatment. The majority of patients who perceived waiting time to be too long expressed the intention of discontinuing treatment^47^ as stated by other studies. Ineffective healthcare systems cause patients’ frustration and result in longer wait times at primary care clinics. Thus, it is imperative that medical facilities measure and shorten patient waiting time, which will result in improved patient experience.^48^

The quality of healthcare services was deemed satisfactory by patients who received subsidised medications in public hospitals in Malaysia, according to a qualitative study.^48^ Thus, provider and client interaction was found to be crucial in helping diabetic patients follow and comply with their treatment regimens.^49^ Diabetes patients in a Zimbabwean study voiced concerns about the compassion, business ethics and communication abilities of healthcare professionals.^50^ Their discontent led them to keep looking for their medications at venues where the costs were reasonable or where their health insurance was accepted, all the while ignoring the calibre of care provided by these establishments.

### Implication for practice

The study findings suggest that the lack of information and misconceptions associated with T2DM and related treatment identified in this study might be addressed through public health campaigns to increase awareness among diabetics and the community at large. Healthcare institutions could craft and implement standard operating procedure (SOP) manuals to guide providers on care counselling and follow-up activities of patients newly diagnosed and those continuing treatment. The SOP might improve communication and patient–provider partnerships to improve and sustain the treatment adherence of diabetic patients.

### Limitations

The study was conducted in only three primary health care facilities, and as such, the findings could not be generalised to the whole of Tshwane district or South Africa. The study’s sample size also limits the generalisability of the findings. By focussing on male patients, the study does not allow for gender comparison and adherence to diabetic treatment. Furthermore, the study did not explore the relationship between other socio-demographic information such as participants’ culture, educational level, marital status and family support and their effects on adherence to treatment. The limitation in demographic information restricts the generalisability of the study findings. Additionally, this study relied on self-report as a method of data collection; therefore, participants’ reports may be vulnerable to recall bias. Despite the highlighted limitations, this study provides evidence of the perceptions of male patients with type 2 diabetes regarding adherence to treatment in clinics in the City of Tshwane, Gauteng province, South Africa.

## Conclusion

Treatment adherence can be enhanced if healthcare providers can address the stigma and myths associated with diabetes mellitus in male patients. By leveraging factors that enhance adherence to treatment, healthcare providers can achieve better treatment outcomes for diabetic patients. It is prudent to embark on community awareness of diabetes mellitus and other non-communicable diseases. In addition, there is a need for family counselling to address stigma and enhance support for the diabetic patient.
